# GPMVs in variable physiological conditions: could they be used for therapy delivery?

**DOI:** 10.1186/s13628-017-0041-x

**Published:** 2018-01-03

**Authors:** Špela Zemljič Jokhadar, Urška Klančnik, Maja Grundner, Tjaša Švelc Kebe, Saša Vrhovec Hartman, Mirjana Liović, Jure Derganc

**Affiliations:** 1 Institute of biophysics, Faculty of medicine, University of Ljubljana, Vrazov trg 2, SI-1000 Ljubljana, Slovenia; 2 Jožef Stefan Institute, Ljubljana, Slovenia; 3 Institute of biochemistry, Faculty of medicine, University of Ljubljana, Ljubljana, Slovenia

**Keywords:** Giant plasma membrane vesicles, Osmotic stress, Lipopolysaccharide, Delivery system

## Abstract

**Background:**

Cell based carriers are increasingly recognized as a good system for cargo delivery to cells. One of the reasons is their biocompatibility and low toxicity compared to artificial systems. Giant plasma membrane vesicles (GPMV) derive from the cell plasma membrane. Thus they offer the closest approximation to it, which makes them good candidates for potential drug delivery systems. To evaluate the applicability of GPMVs as carriers, we analyzed their basic biophysical properties to test their robustness in the face of changeable physiological conditions, as well as their ability to translocate across the membrane into cells.

**Results:**

GPMVs formed from human umbilical vein endothelial cells (HUVEC) sustain a drastic osmotic challenge (50–500 mOsmoL/kg) unlike giant unilamelar vesicles (GUVs). In hyper-osmotic solutions the average volume decreases and membrane invaginations form, while in the hypo-osmolar buffer the volume of GPMVs increases and these changes were not reversible. The membranes of flaccid GPMVs started to wrinkle unevenly giving rise to buds after exposure to lipopolysaccharide (LPS). The shape changes in GUVs are reversible in contrast to GPMVs after LPS removal. GPMVs exposed to fluorescent LPS exhibited a signal that remained visible in some GPMVs even after LPS removal, which was never the case with GUVs. Calcein penetrated both into GUVs and GPMVs, however after the removal from the bulk solution some of the GPMVs still exhibited very bright signal, while in GUVs only a weak fluorescent signal was detected. We could also see that practically all GPMVs incorporated dextran initially, but after the dextran solution was changed with the initial non-fluorescent solution it remained only in 20% of them. The majority of HUVEC cells displayed a fluorescent signal after the incubation with GPMVs that contained fluorescently labeled dextran.

**Conclusion:**

Our findings indicate that GPMVs behave quite differently from artificially made giant phospholipid vesicles and the changes induced by the different treatments we subjected them to are not reversible. We also demonstrate that different substances can be both loaded into them and delivered into cells, so GPMVs may be of potential use as cargo/therapy delivery systems.

## Background

Liposomes are artificial vesicles consisting of single or bilayer membranes encapsulating an aqueous compartment [1]. Unique physical properties that mimic biological membranes made them useful in a broad range of scientific and biological applications [2]. The size and lipid composition of vesicles can differ considerably and the choice of the components determines the fluidity and rigidity of the bilayer [1]. Nevertheless, the structure of lipid vesicles is relatively simple compared to the complexity of the cell membrane, and the knowledge gathered from this model system is limited [3]. On the other hand giant plasma membrane vesicles (GPMVs) can be derived from different types of cells by chemically induced plasma membrane vesiculation or »blebbing« [4]. In addition they are of similar size to the synthetically made giant unilamelar vesicles (GUVs), but retain the composition of the plasma membrane (PM). Furthermore, they lack the complex structures present in the cytosol and the interactions between the membrane and the cytoskeleton [5, 6], which was exploited already in early eighties in a study of changes in diffusibility of membrane components [7]. Despite the deficiencies arising from the chemical modification required for their isolation, GPMVs offer the closest approximation to the PM [6]. Altogether this makes them a very good model system to study composition, properties and functions of the cellular PM [8].

GPMVs gained importance after Baumgart et al. [4] observed a liquid –liquid phase separation in their membranes, which depends critically on membrane cholesterol [9]. These findings support the main concept of the raft hypothesis: the capacity of biological membranes to separate into coexisting fluid phases of distinct composition and physical properties [8]. Thus the majority of research on GPMVs engages studies on coexisting fluid phases [4, 5, 9–12]. However the unique membrane composition of GPMVs could be an asset in studies of membrane properties, which were traditionally done on GUVs [13]. In fact, due to their properties GPMVs appear to have great potential for a broad range of applications.

One possibility would be to use GPMVs as drug carriers, and some studies already tested natural cells or cell-derived vesicles for this purpose [14, 15]. Artificially made liposomes are widely used for delivery of therapeutics, however their lipid composition could also result in some adverse effects [3]. On the other hand a carrier with the cell membrane’s intrinsic composition may demonstrate better biocompatibility and lower toxicity. GPMVs were already used as a model to study the translocation of cell-penetrating peptides across the plasma membrane without the interference of endocytotic processes [16, 17], and it was also shown that amphiphilic quantum dots can penetrate GPMVs’ membrane [18].

However before any strategies involving GPMVs as carriers can be developed, basic biophysical characteristics should be evaluated in order to determine whether they are able to withstand the variable physiological conditions in the human body. Beside this, the ability of different substances to translocate across the GPMVs membrane should be also carefully evaluated. As GPMVs size is close to GUVs, i.e. are visible under an optical microscope, we can visually inspect these vesicles for membrane changes. This technique has already been well implemented in the GUVs field of research. In addition, the similarity in size and shape of both membrane systems enables us to do a direct comparison between them, and consequently also allows to expand the existing knowledge on the effects of osmotic challenge or lipopolysaccharide (LPS) exposure on GUVs [19].

Taking all this under consideration, in this study we decided to focus on: a) The effects of osmotic challenge on GPMVs membrane; b) The outcome of adding LPS, a physiologically relevant amphiphilic molecule, which is the main component of the Gram negative bacteria cell wall [20]; and c) The permeability of the GPMV membrane to non-specific substances such as calcein AM and Alexa Fluor conjugated dextran. The presented data is promising, as it indicates GPMVs can be easily produced and may have a potential application for drug delivery.

## Methods

### The reagents

1,2-dioleoyl-sn-glycero-3-phosphocholine (DOPC), sphingomyelin (SM, brain porcine), cholesterol (Chol) and 1-palmitoyl-2-(6-((7-nitro-2-1,3-bezoxadiazol-4-yl)amino)hexanoyl)-*sn*-glycero-3-phosphocholine (NBD-PC) were purchased at Avanti Polar Lipids (USA). LPS from *E.coli* (serotype O55:B5) and FITC conjugated LPS from *E. coli* (serotype 0111:B4) as well as AMP-PNP ((adenylyl-imidodiphosphate) were purchased at Sigma Aldrich. Osmolality was continuously measured throughout the experiment with an osmometer Semi-micro K-7400 (Knauer, Germany).

### Preparation of phase segregated giant unilamellar vesicles (GUVs)

We prepared a phospholipid mixture of 1 mM DOPC, 1 mM SM and 1 mM Chol (volume ratio, 32.5:32.5:35) with 1 vol.% of 1 mM NBD-PC as a fluorescent marker of the liquid disordered phase. The lipids were dissolved in a mixture of chloroform-methanol (volume ratio, 2:1). We applied 25 μL of the lipid mixture onto an electrode made of inert platinum and left it to dry in vacuum at room temperature. GUVs were formed with electro-formation in a vial filled with 200 mM sucrose solution at 58 °C according to Angelova et al. [21]. The vesicles were stored in 200 mM glucose solution up to 3 days in sealed test-tubes at room temperature prior to use.

### Preparation of giant plasma membrane vesicles (GPMVs)

For all the experiments concerning GPMVs, they were isolated from human umbilical vein endothelial cells (HUVEC; ATCC) using a slightly modified method of Scott [22]. Cells were grown to ~ 70% confluence in 25 cm^2^ tissue culture flasks before rinsing with GPMV buffer (150 mM NaCl, 2 mM CaCl_2_, 10 mM HEPES, pH 7.4). Cells were incubated with a mixture of 25 mM paraformaldehyde and 2 mM dithiothreitol in GPMV washing buffer for 2 h at 37 °C in CO_2_ incubator to vesiculate. GPMVs were collected and left in the GPMV buffer at room temperature for 30 min before use. The liquid disordered phases of GPMVs were marked with a fluorescent marker, β-BODIPY FL C5-HPC (Molecular Probes) [23]. 1.5 vol.% of the marker was added to the vesicle suspension in GPMV buffer, and left to slowly rotate for 10 min at room temperature.

### Shape change monitoring and image analyses

Only spherical GUVs and GPMVs that showed no membrane protrusions were loaded into the microfluidic diffusion chamber using optical tweezers (Aresis d.o.o., Slovenia). The microfluidic device, which allows a controlled exchange of liquid environment without disturbing the vesicles themselves, was set up as described in Vrhovec et al. [24]. Shape changes were monitored under a Nikon Eclipse Ti inverted microscope with Andor Zyla sCMOS camera and images acquired with the MicroManager 1.4.18 program. Each set of experiments involved 4 to 7 vesicles and was repeated 3 to 5 times independently.

Microscopy images and video clips were analyzed using ImageJ software. The radii of the vesicles were measured using the oval measure function and averaged over three independent measurements for each point in time. Due to the distorted geometrical shape of the vesicles when exposed to LPS, only the beginning and end radii were measured, when the vesicles were in their initial buffer. The differences were analyzed using Student’s *t*-test on 2 populations and one-way ANOVA; *p* < 0.01 was considered significant.

### Osmolality

The initial osmolality of GPMVs suspension was 300 mOsmoL/kg and this was gradually changed during the experiments with hyper-osmotic solutions in two consecutive 5 min steps (first to 400 and then to 500 mOsmoL/kg), after which they were reversibly reduced, again with two 5 min intergraded steps (first to 400 than to 300 mOsmoL/kg). In experiments with hypo-osmotic solutions the initial suspension was changed to 50 and then back to 300 mOsmoL/kg. GPMVs responses were monitored all the time.

### Lipopolysaccharide

The LPS solution was prepared directly from dehydrated powdered form, without further purification. LPS powder was dissolved in the respective buffer (glucose solution or GPMV buffer) directly before use at a concentration of 10 μg/mL, which is on the limit of the critical micellar concentration for *E.coli* LPS [25]. FITC conjugated LPS was dissolved in phosphate buffered saline to get a bulk solution (1 mg/mL), from which 10 μg/mL working solution was prepared. LPS solutions were kept at room temperatures during the experiments.

The initial respective buffer (glucose solution or GPMV buffer) was changed to a buffer of 10% higher osmolality to make the vesicles more flaccid and the shape changes more pronounced. After the vesicles became flaccid, the solution was changed to 10 μg/mL LPS solution (at higher osmolality).

The LPS solution was replaced with high osmolality buffer after ~ 8 min of LPS exposure. After 2 to 3 min, as the LPS were removed from the diffusion chamber, the high osmolality buffer was washed with the initial buffer. FITC conjugated LPS was used to examine LPS binding to the vesicles in fluorescent confocal mode of microscope. The vesicles in the buffer with higher osmolality without LPS were used as the control.

### Calcein AM and Dextran loading

Both types of vesicles were incubated in Calcein AM (Thermofisher) solution (1:100) or Alexa Fluor conjugated dexstran with the molecular weight 10,000 (Thermofisher) for 30 min in glucose for GUVs, or GPMV buffer for GPMVs. Thereafter both calcein AM and dextran were either washed away in the microfluidic chamber or purified by repeated centrifugation (for GPMVs), after which the vesicles were imaged. In some cases vesicles were imaged even before calcein or dextran was washed out with the fluorescent microscope in epifluorescence or confocal mode.

### The delivery of dextran from GPMVs into HUVEC cells

HUVEC cells were grown in the serum reduced minimum essential medium (MEM) (Gibco) supplemented with fetal bovine serum (Gibco) and antibiotics (streptomycin-penycilin) (Gibco). One day prior the conductance of the experiments the cells were seeded (30.000 cells/ml) in a petri dish (35 mm diameter) with a glass bottom (Ibidi, Germany).

The GPMVs loaded with dextran were performed as described previously with an additional step in the procedure. After GPMVs were harvested from the cells and before dextran loading the vesicle suspension was thoroughly washed by repeated centrifugation. The loaded GPMVs were then added to the HUVEC cells and incubated for 24 h before a visual inspection on the microscope.

## Results

### Osmotic stress

**Fig. 1:**
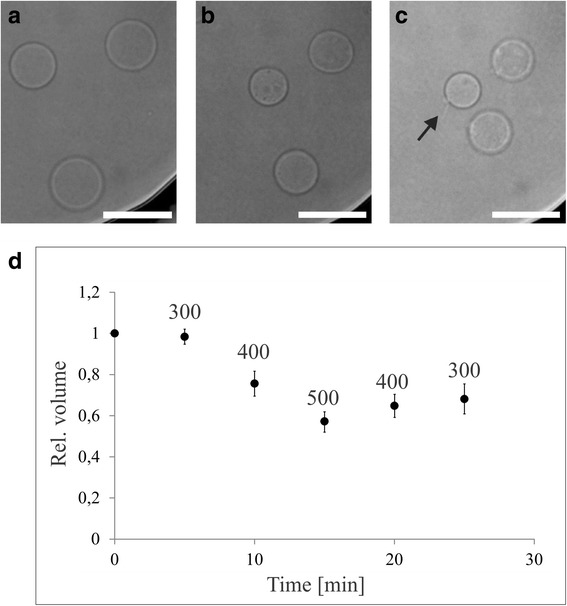
The effects of a hyper-osmotic solution on GPMVs. **a** The GPMVs were first kept in an isosmotic solution (300 mOsmoL/kg) and then **b** the solution was changed to a hyper-osmotic (500 mOsmoL/kg and **c** again to the initial one, where evaginations formed from some of them (see arrow). The GPMVs were imaged under an inverted microscope in bright-field with a water immersion objective (magnification 60X). **d** Time course of average GPMV volume change (*n* = 17). Between 0 and 5 min the vesicles were in the isosmotic solution (300 mOsmoL/kg), which was then gradually changed first to 400 mOsmoL/kg and after 5 min to 500 mOsmoL/kg. After 5 min the solutions were again gradually changed to decrease the osmotic value to 300 mOsmoL/kg at the end. The bar represents 10 μm

To test their robustness, we exposed the GPMVs to solutions with a broad range of osmotic values (from 50 to 500 mOsmoL/kg). In the first set of experiments, the initial solution was changed to a hyper-osmotic solution (Fig. 1). GPMVs, formed in the isosmotic solution (300 mOsmoL/kg) from HUVECs, were of different sizes (5–15 μm radius) and had a spherical, slightly fluctuating shape (Fig. 1a). After the osmolality of the solution was changed to 400 mOsmoL/kg, the GPMVs shrank, retained the spherical shape and formed invaginations in the form of small buds (Fig. 1b). The shrinkage progressed when the osmolality was changed to 500 mOsmoL/kg after 5 min. Finally, after the osmolality was gradually changed back to 300 mOsmoL/kg, the vesicles inflated to a size that was smaller than the initial one and the internal protrusions remained visible (arrows on Fig. 1c). The volumes of GPMVs during the experiment were quantified from the measured vesicle radius (Fig. 1d). We found that the average change in volumes followed the increase in osmolality: at 400 mOsmoL/kg the average volume was 75% of the initial one, and at 500 mOsmoL/kg the volumes shrank to 57%. However these changes were not reversible upon reversal to initial conditions: 65% at 400 mOsmoL/kg and 68% at 300 mOsmoL/kg.

**Fig. 2:**
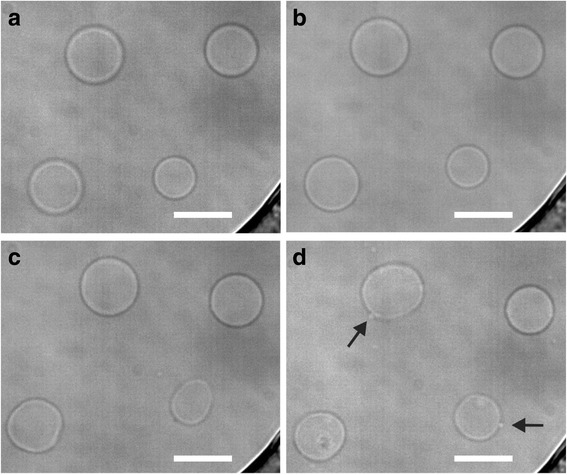
The effects of a hypo-osmotic solution on GPMVs. **a** The GPMVs were first kept in an isosmotic solution (300 mOsmoL/kg). The solution was then changed to a hypo-osmotic solution and **b** the membrane became tense. After approximately 3 min **c** the membrane started to fluctuate. The solution was changed back to the isosmotic **d** again, the fluctuation remained and evaginations formed in some GPMVs (see arrows). The GPMVs were imaged under an inverted microscope in bright-field with a water immersion objective (magnification 60X). The bar represents 10 μm

In the second set of experiments the isosmotic solution was changed to hypo-osmotic solution (50 mOsmoL/kg) and then back again (Fig. 2). After the addition of the hypo-osmotic solution the vesicle membrane tightened (Fig. 2a) and the relative volume increased approximately 5% on average (Fig. 2b). This quickly changed within a few minutes of exposure, and the GPMVs became increasingly flaccid and started to fluctuate (Fig. 2c). When the vesicles were returned to the isosmotic solution they still retained the flaccid shape and some even formed protrusions (arrows on Fig. 2d). Volume quantification could not be performed for the fluctuating non-spherical vesicles.

### Lipopolysaccharide

In this set of experiments we exposed both GUVs and GPMVs to LPS, an amphiphilic molecule that is crucial for gram-negative bacterial infections.

**Fig. 3:**
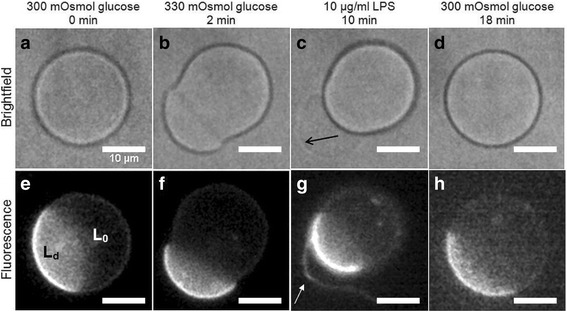
LPS caused the formation of vesicle-like evaginations and tethers from the l_d_ phase of GUVs. Shape change of a representative GUV in a microfluidic diffusion chamber under bright field (**a**-**d**) and the same vesicles under fluorescence. The l_d_ phase was marked with NBD-PC marker. Initially, the osmolality of the surrounding buffer was changed from 300 to 330 mOsmoL/kg, making the vesicle more flaccid (**a, e**). After 8 min, 10 μg/mL LPS was added and tether-like evaginations developed (**b**, **c**, **f**, **g**). The arrows in images c and g point at a tether. When LPS was replaced with LPS-free solution, GUV occupied their original shape (**d**, **h**). The GUVs were imaged under an inverted microscope in bright-field and epi-fluorescence mode (magnification 60X). The bar represents 10 μm

Once GUVs were secured in the diffusion chamber, the initial glucose solution was switched to a solution of higher osmolality prior to the addition of LPS (Fig. 3a, e). The membranes became looser and the shape change was easier to observe. In fact, when the membrane was not flaccid enough, no LPS induced shape change could be observed. The presence of 2 phases (liquid ordered - l_o_ and disordered - l_d_) was examined with the NBD-PC marker that binds to the l_d_ phase (Fig. 3e-h). Adding 10 μg/mL solution LPS to flaccid GUVs resulted in the formation of small vesicle-like evaginations (referred to as buds) and tethers (arrows on Fig. 3c and g) mainly from the l_d_ phase of the GUVs, which could be observed by fluorescence microscopy (Fig. 3g). After LPS was removed from both solutions the GUVs returned to their original shape as well as the shape change proved to be totally reversible within 2 min of LPS removal (Fig. 3d, h). The phases of GUVs did not merge or relocate. In addition, during the entire time LPS was present in the surrounding medium the l_o_ phase appeared without any fluctuations or shape changes. The difference in the radius size before and after LPS exposure was not statistically significant, amounting to ±3%. To avoid possible changes due to osmotic pressure, measurements were done when vesicles were in their respective initial buffers.

**Fig. 4:**
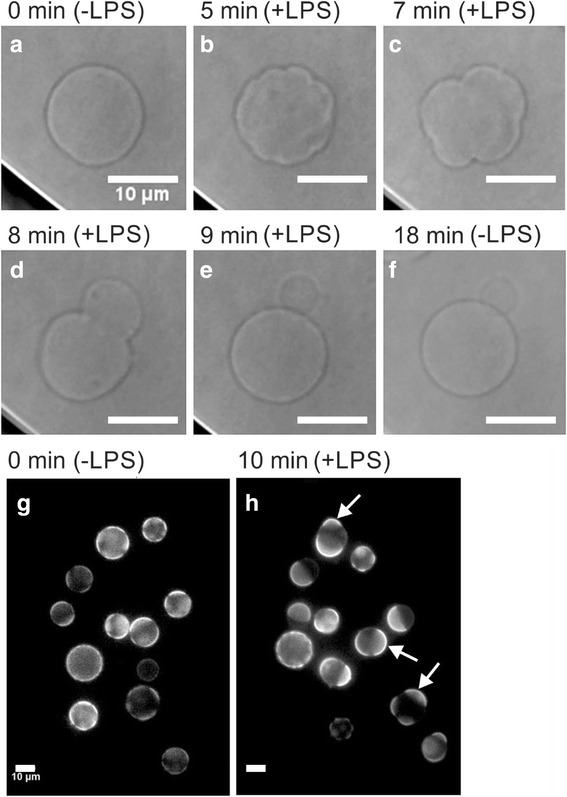
The addition of LPS caused wrinkling of the GPMVs membrane and formation of buds. Shape change was observed under bright field illumination after exposure to 10 μg/mL LPS. Initially the osmolality of the surrounding buffer was increased from 300 to 330 mOsmoL/kg making the vesicles more flaccid (**a**). GPMVs 4 (**b**), 5 (**c**), 7 (**d**) and 8 min (**e**) after adding LPS. The vesicle started to fluctuate, bulge and form a bud, which remained after LPS was removed from the buffer (**f**). With epi-fluorescence microscopy we could see that GPMVs bulge from l_d_ phases after the addition of LPS. GPMVs marked with β-BODIPY FL C5-HPC before LPS addition (**g**) and 10 min after the addition of LPS solution at 10 μg/mL, bulging of the l_d_ phase was observed (**h**). Some phase merging could also be seen. The GPMVs were imaged under an inverted microscope in bright-field and epi-fluorescence mode (magnification 60X). The bar represents 10 μm

After exposure to LPS membranes 48% of flaccid GPMVs (Fig. 4a) started to wrinkle unevenly (Fig. 4) giving rise to buds (Fig. 4e, f). The difference in radius size before and after LPS exposure was (as in GUVs) not statistically significant (±3%).

After the addition of BODIPY fluorescent l_d_ phase marker [23] (Fig. 4g, h), we could also observe GPMVs with fluorescence microscopy. Initially circular, flaccid GPMVs (Fig. 4g) started to bulge from l_d_ phases after the addition of LPS (Fig. 4h white arrows). Some parts bulged more than others, which was similar to what was observed on GUVs, indicating that the membrane consisted of different phases, just as previously described [11]. In some cases, when LPS was added to GPMVs the phases relocated and merged into larger poles. GPMVʼs membranes fluctuated and formed new evaginations even after 50 min exposure to LPS.

**Fig. 5:**
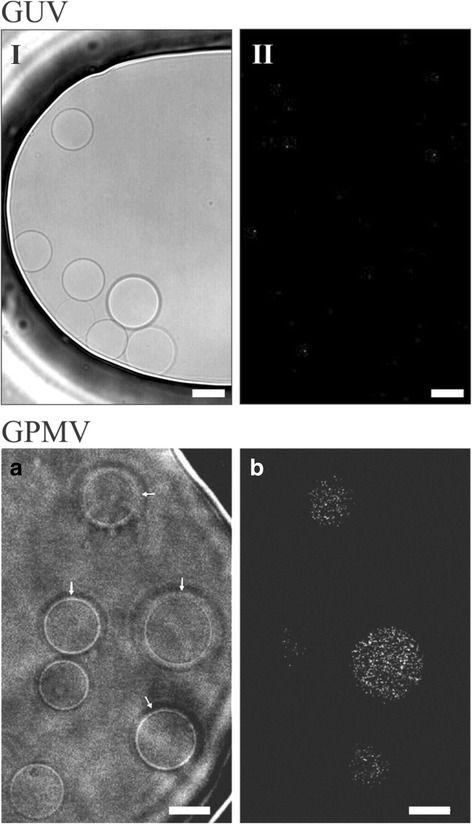
When LPS was replaced with LPS-free solution, GUV occupied their original shape as seen on the bright-field image (**I**) and no fluorescent signal could be detected (**II**). In GPMVs the signal from fluorescent LPS remained in the vesicles after LPS removal. GPMVs exposed to 10 μg/mL FITC conjugated LPS in the microfluidic diffusion chamber for 10 min. The surrounding LPS solution was then switched to GPMV buffer without LPS (**a**). The bound LPS signal was visible in some vesicles (arrows) even after the depletion of LPS from the solution (**b**). The vesicles were imaged with a confocal microscope (magnification 60X). The bar represents 10 μm

An important difference between GUVs and GPMVs is that the changes in shape of the latter are irreversible, even after LPS removal (Figs. 4f and 5a). Also, GPMVs exposed to fluorescent LPS exhibited a signal that remained visible in some GPMVs even after LPS removal (Fig. 5b), which was never the case with GUVs. There the shape changes were reversible, but no signal from fluorescent LPS could be detected (Fig. 5I, II). Furthermore, the signal was inside the vesicle so the labeled LPS must have had translocated across the membrane into the vesicle lumen.

### Permeability of the GPMVs membrane

The permeability of the GPMVs versus GUVs membranes was tested by adding calcein AM or fluorescently labeled dextran (10,000 MW) to the solution.

**Fig. 6:**
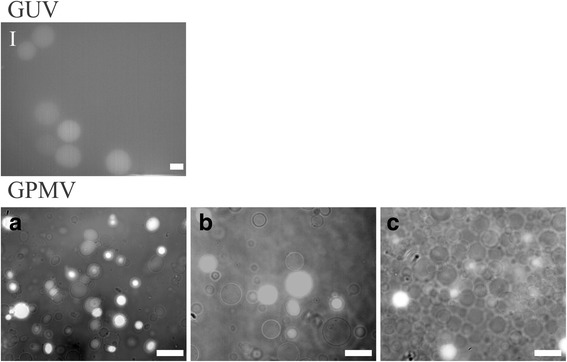
Calcein AM translocate into GUVs and remained there also after it is removed from the bulk solution, but the fluorescent signal was relatively weak (**I**). Fluorescent signal representing calcein (**a**) or Alexa Fluor labeled dextran (**b**) remained intensive after the agents were removed from the bulk solution by repeated centrifugation. Inhibition of ATP hydrolysis caused a markedly increased formation of GPMVs, however the incidence of dextran positive vesicles (brighter vesicles) was low (**c**). GPMVs were imaged both in bright-field and epi-fluorescent mode and the images were then merged. The objective magnification was 60×. The white bar represents 10 μm

**Fig. 7:**
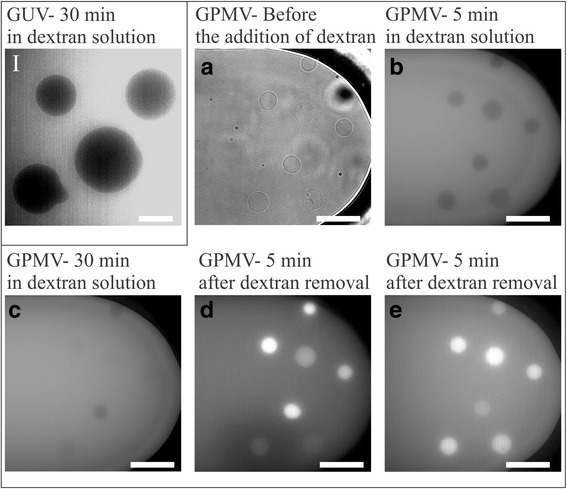
GUVs did not incorporate any Alexa Fluor labeled dextran as seen on a fluorescent image (**I**). Alexa Fluor labeled dextran translocates into most of the GPMVs, however it does not stay in all of them after the wash out. **a** GPMVs in a microfluidic chamber before the addition of dextran imaged in bright field. **b** GPMVs 5 min and **c** 30 min after the addition of dextran solution imaged in epi-fluorescence mode. **d** GPMVs 5 min and **e** 30 min after the dextran solution was changed with the initial one imaged in epi-fluorescence mode. The objective magnification was 60×. The white bar represents 10 μm

Calcein AM is an uncharged molecule that can permeate cell membranes. The permeability experiments were performed in the microfluidic chamber. Within a few minutes of calcein addition we could not distinguish any more the vesicles from the surrounding solution, as calcein penetrated both into GUVs and GPMVs. However after this solution was replaced with the initial non-fluorescent solution, the GUVs exhibited a very weak fluorescent signal (Fig. 6I) indicating that calcein has diffused out, while the fluorescent signal in a part of GPMVs still remained intensive. GPMVs exhibiting a bright fluorescent signal were also seen in the bulk suspension (Fig 6a). The membrane permeability was further tested with dextran, a relatively large molecule that cannot penetrate the membrane of synthetically made vesicles, as demonstrated also in our experiments with the microfluidic chamber (Fig. 7I). Once again GPMVs behaved differently in comparison to GUVs. While dextran translocated into some of them, some dark spots were still observed, indicating that some GPMVs did not incorporate any dextran. Inspection of the vesicles in the bulk solution revealed that after several washings the fluorescent signal was still detectable in 20% (146 out of 731 GPMVs) of the vesicles (Fig. 6b). If ATP hydrolysis in cells is inhibited by the non-hydrolysable ATP analog AMP-PNP (adenylyl-imidodiphosphate), the number of obtained GPMV vesicles increases (7×), but the occurrence of dextran positive GPMVs was relatively low (2%) compared to untreated GPMVs (20%) (Fig. 6c).

We secured the GUVs and GPMVs in the microfluidic diffusion chamber to study the dextran intake in time lapse (Fig 7). GUVs were seen as dark circles on a bright background (Fig. 7I) even 30 min after fluorescent dextran was introduced in the microfluidic chamber, indicating that no dextran translocate into them. GPMVs were initially in GPMV buffer (Fig. 7a), which was replaced with the GPMV buffer that contained fluorescent dextran. We could see that practically all GPMVs incorporated dextran (Fig. 7b, c), but after the dextran solution was changed with the initial non-fluorescent solution again (Fig. 7d, e) it vanished from some of them.

### The ability of GPMVs to deliver dextran to cells grown in vitro

**Fig. 8:**
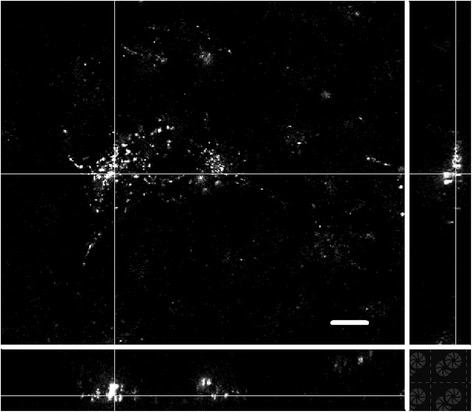
GPMV delivered fluorescence dextran in HUVEC cells. Dextran is present in most HUVEC cells after they were incubated for 24 h with dextran loaded GPMVs. These GPMVs were also formed from HUVEC cells to allow higher biocompatibility. The HUVEC cells were imaged with a confocal microscope and the image is composed of the top view (in the middle) and side views (bottom and right) of dextran loaded cells (magnification 60X). The white bar represents 10 μm

After GPMVs production the suspension of vesicles was thoroughly washed so the potentially toxic chemicals needed for GPMV formation would not interfere with cell growth. This was additionally confirmed by testing cell viability after the addition of such GPMVs to cells grown in culture. Two days after the addition of vesicles the relative cell viability (compared to the control) was still high, 92 ± 4.4%. The HUVEC cells were incubated for 24 h with fluorescent dextran loaded GPMVs in cell medium and visually inspected afterwards on the microscope. The majority of cells displayed a fluorescent signal, which may be interpreted as presence of dextran in their interior (Fig. 8).

## Discussion

Cell membrane based carriers demonstrate better biocompatibility and lower toxicity than liposomes [3]. A promising formulation used for drug delivery are exosomes [26, 27] and also whole cells, from which red blood cells are most frequently used [3]. Amongst their greatest advantages are the natural surface that can protect the encapsulated cargo from inactivation and its long lifespan. The intrinsic membrane properties of GPMVs make them, at least in theory, a realistic drug delivery system. Until now the majority of data obtained on GPMVs regards membrane rafts, while only a few studies focused on the permeability of GPMVs membrane [16, 18]. The ability of different molecules/substances to translocate into the lumen and the capability of vesicles to adapt to variable conditions, are important parameters for any prospective carrier system.

In some of our experiments we compared the results assessed on GPMVs with the effects on GUVs, as the basic biophysical features assessed on GUVs are well characterized. To mimic as close as possible the GPMV membrane composition, we here used phase segregated GUVs. We found that GPMVs are a robust system as they can sustain handling procedures like centrifugation and sonication, which is not the case with GUVs.

Dehydration as a consequence of disease can lead to changes of extracellular body fluids tonicity (blood, interstitial fluid). In order to test this we exposed GPMVs to solutions with a broad range of osmotic values. Interestingly, GUVs do not sustain such a drastic osmotic challenge, demonstrating that GPMVs are significantly more robust. In hyper-osmotic solutions the water leaves the lumen as anticipated, consequently reducing the relative volume of vesicles, while the membrane area stays the same giving rise to membrane invaginations (Fig. 1). When applying the hypo-osmolar buffer GPMVs initially swell due to the pressure caused by the water influx to a point where the water pressure stretches the membrane to its limits (Fig. 2a, b). The tensed membrane than abruptly became flaccid (Fig. 2c) as transient pores form through which the solutes can escape and the pressure on the membrane diminishes. After the relaxation, the membrane closes and the osmolality increases, that causes the formation of evaginations in some GPMVs (Fig. 2d, arrows). Comparable results, however with only slightly changed osmotic gradients on phase segregated GUVs were reported by Oglecka et al. [28]. The changes on the GPMVs membrane were irreversible after the osmotic challenge regardless of osmotic gradients.

Lipid A, the hydrophobic part of LPS that is responsible for biological toxicity [29], inserts into the membrane of GUVs from the aqueous solution and causes pronounced membrane deformations [19]. As seen in our experiments this is also the case with GPMVs. We used the LPS from *E.coli* that has an inverted cone 3D shape [30, 31]. Because of this shape it presumably acts as a wedge when inserted into the outer vesicle membrane. Additionally LPS molecules are too large to flip-flop to the inner membrane monolayer. Thus their insertion into the membrane increases only the surface area of the outer membrane monolayer and imposes vesicle budding by the bilayer-couple mechanism [19]. According to the area difference elasticity (ADE) model of vesicle shapes, only a 0.1% increase of the surface area of the outer layer of a flaccid vesicle can produce marked outer membrane protrusions and budding [32]. Such shape transformations have already been observed in GUVs composed of either l_o_ or l_d_ lipid phases [33], and now we could detect them also in phase segregated GUVs and GPMVs. In both vesicle types we observed protrusions and bulges emerging predominantly from the l_d_ phase (Figs. 3 and 4). This is in agreement with previous studies showing that l_d_ phase is less rigid and so easier subjected to deformation than the l_o_ phase [34]. The two membrane phases are mechanically coupled in the lateral direction, and according to the ADE model the protrusions emerging from the l_d_ phase relax the stress in the outer leaflet of the l_o_ phase, too. Importantly, all shape changes of GUVs were reversible upon removal of LPS from the microfluidic diffusion chamber, indicating nonspecific and reversible binding of LPS to the membrane (Figs. 3 and 5I)), something that is in agreement with Alam and Yamazaki [19]. This is supported also by the notion that we could not follow LPS binding after adding fluorescently conjugated LPS to them, as they were washed out as soon as the surrounding solution was changed (Fig. 5II). In contrast to GUVs, the GPMVs did not relax back to the initial shape after LPS removal (Fig. 4). Also, in some of them a weak fluorescent signal could be observed after fluorescently labeled LPS was removed from the bulk (Fig. 5b). This indicates that LPS translocated into GPMVs lumen, although it is supposed to be too large to flip-flop to the inner membrane monolayer, which was demonstrated in the GUVs experiments.

The ability of translocation through the membrane of GPMVs was tested with two different substances, Calcein AM and fluorescently labeled dextran (MW 10.000), which may be also used to study drug delivery vehicles [35]. Calcein is also commonly used as an indicator of lipid vesicles leakage [36]. While in most experiments Calcein AM is trapped inside the vesicles during their formation, we on the other hand added it to already formed vesicles. After washing, only weak fluorescent signal remained in GUVs (Fig. 6I) in comparison to GPMVs, where a bright fluorescent signal was detected (Fig. 6a). This can be due to the fact that GPMVs lumen is filled with cytosol and therefore also contains Ca^2+^ and nonspecific esterases that cleave the AM group and trap it inside GPMVs. However not all vesicles contain all the factors needed to successfully entrap Calcein, and so in some vesicles we could not observe any fluorescent signal after washout (Fig. 6a).

Different cells can take up dextran preferably via specific receptors but also by mechanisms of nonspecific fluid-phase endocytosis [37]. This means it cannot freely pass into vesicles like Calcein, which could be also seen with our GUVs (Fig. 7I). Saalik et al. [16] reported that dextran also did not translocate from the medium into the lumen of GPMVs. Nevertheless in our experiments we could clearly see that dextran translocates into some GPMVs and also stays there after it is washed from the bulk solution (Fig. 7), perhaps due to some type of transport across the membrane. To test if this transport is ATP dependent, we treated cells with AMP-PNP prior to GPMV formation. We found that much more (>100%) GPMVs form in treated compared to untreated cells. The reason is that due to inhibition of ATP dependent processes the cells turn to apoptosis, which increases membrane blebbing. When these GPMVs were loaded with dextran, fluorescent signal was detected in fewer GPMVs than in vesicles formed from untreated cells. The transport into the vesicles lumen does not seem to be ATP dependent or at list not completely. However it can be expected that the membrane of some GPMVs contain specific receptors that may assist dextran intake into cells.

To test the idea of cargo delivery, GPMVs with fluorescently labeled dextran were incubated with cells grown in vitro. Amongst these we found that most of the cells displayed some fluorescence (Fig. 8). The yield of fluorescent cells was low, but we think that the procedure could be improved further so the effectiveness could be much higher.

## Conclusion

To summarize our findings, GPMVs are very diverse in their membrane composition. They behave differently than GUVs, in particular they are more robust. Another important characteristic of GPMVs is that the shape changes that occurred during our experiments were not reversible regardless of the challenge (osmotic stress, the addition of amphipathic molecule - LPS). LPS and dextran translocation into the vesicle lumen indicates that membrane proteins remain in GPMVs membrane after they form. We also showed that we can load nonspecific cargo into formed GPMVs and that they may fuse with cells as the cargo is transferred into them. Based on this we believe that GPMVs are potentially useful drug delivery systems, but further investigation is needed to optimize this system and to increase its efficiency.

## Abbreviations

ADE: Area difference elasticity
GPMV: Giant plasma membrane vesicles
GUV: Giant unilamelar vesicles
l_d_: Liquid disordered
l_o_: Liquid ordered
LPS: Lipopolysaccharide
PM: Plasma membrane
